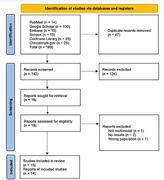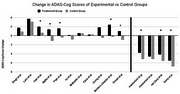# Multi‐Modal Lifestyle Interventions vs Anti‐amyloid Medications for Patients with MCI or Early‐Stage Alzheimer’s Disease; Which One Is More Effective?

**DOI:** 10.1002/alz70861_108708

**Published:** 2025-12-23

**Authors:** Majid Fotuhi, Sidharth Pavulri, Mikhail Coen

**Affiliations:** ^1^ Johns Hopkins University, Baltimore, MD USA

## Abstract

**Background:**

Late‐onset Alzheimer’s disease (AD) is driven by multifactorial pathology, necessitating treatments that address multiple mechanisms. Multimodal lifestyle interventions (combining exercise, diet, cognitive training, etc.) have shown promise for patients with mild cognitive impairment (MCI) and early‐stage AD. Meanwhile, anti‐amyloid monoclonal antibodies (e.g., aducanumab, lecanemab, donanemab) have demonstrated modest efficacy in slowing cognitive decline in early AD. However, their comparative real‐world cognitive benefits and cost‐effectiveness versus lifestyle interventions remain unclear.

**Method:**

A systematic review identified 11 randomized controlled trials of lifestyle‐based multi‐domain interventions and Phase 3 trials of anti‐amyloid monoclonal antibodies. Key cognitive outcomes (especially ADAS‐Cog) were qualitatively compared across studies, and reported intervention costs were noted.

**Result:**

Multimodal lifestyle interventions consistently produced cognitive benefits in patients with MCI or early AD. Six of eleven lifestyle trials reported statistically significant ADAS‐Cog improvements in intervention groups versus controls, and nine showed improvement in at least one cognitive measure. Participants receiving lifestyle interventions often exhibited not just stabilized but improved cognitive scores over 6–12 months, alongside broader health gains (e.g., improved cardiovascular fitness and mood). In contrast, monoclonal antibody treatments significantly **slowed** the rate of cognitive decline but did not demonstrate actual **improvements** in cognitive test scores or noticeable symptom relief. Furthermore, lifestyle approaches were substantially less expensive per patient (on the order of a few thousand USD annually) compared to high‐cost monoclonal infusions ($50,000 to $70,000), and lifestyle interventions carry minimal risk relative to drug‐related adverse events.

**Conclusion:**

For patients with MCI or early AD, multimodal lifestyle interventions offer meaningful cognitive improvement along with safety and cost advantages, making them a compelling therapeutic option. While monoclonal antibodies provide only a modest slowing of cognitive deterioration at significant financial cost and risk, lifestyle‐based approaches may be a safer and more cost‐effective strategy to preserve cognitive function. These findings underscore the need for larger‐scale trials and head‐to‐head comparisons to guide clinical practice.